# Married Health Visitors

**Published:** 1920-10-02

**Authors:** 


### Married Health Visitors.

The question as to whether married women should hold appointments has been raised by the General Purposes Committee of the Lambeth Borough Council. Miss Theresa McHugh was appointed a health visitor in 1910. When war broke out her fiancé was ordered abroad, and she obtained permission from the Public Health Committee to get married and retain her position during the war. Dr. Priestley, the Medical Officer of Health, in a report to the committee, spoke very highly of the way in which Nurse McHugh had carried out her duties, and was of opinion that it would be a distinct loss to the health administration of the borough if her services were dispensed with. The General Purposes Committee, however, thought that the time had now arrived when she should be asked to resign her office in favour of a lady who is unmarried or a widow. The council would not endorse this, and by an overwhelming majority are in favour of retaining the services of married ladies on the staff. The matter is one which deeply concerns the future of public health nursing. The hours are now generally limited to eight, on six days only in the week, and there is no reason why a woman should not carry out the duties as efficiently after marriage as before. Health visitors are not made in a moment. Their training, when begun, as it should be, in hospital, lasts fully four and often five years, while the ripe experience which goes to make them a real power in their district is not attained for two more. Why should all this be thrown away on their marriage? There is far too heavy a wastage already through the married nurses who abandon the practice of their profession. As regards private or institutional nursing this may be considered inevitable. But public health visiting positively asks for married women to carry it out. Their appeal is to the married, and young health visitors called to advise the matrons of the place about their difficulties are at a grave disadvantage as compared with their married *confrères*. Well done, Lambeth!

